# Uniaxial Magnetization and Electrocatalytic Performance for Hydrogen Evolution on Electrodeposited Ni Nanowire Array Electrodes with Ultra-High Aspect Ratio

**DOI:** 10.3390/nano14090755

**Published:** 2024-04-25

**Authors:** Yumu Sako, Ryusei Saeki, Masamitsu Hayashida, Takeshi Ohgai

**Affiliations:** 1 Graduate School of Engineering, Nagasaki University, 1-14 Bunkyo-machi, Nagasaki 852-8521, Japan; 2 Graduate School of Engineering, Kyushu University, 744 Motooka, Nishi-ku, Fukuoka 819-0395, Japan; 3 Faculty of Engineering, Nagasaki University, Bunkyo-machi 1-14, Nagasaki 852-8521, Japan

**Keywords:** anodization, aluminum, nanochannel, electrodeposition, nickel, nanowire, magnetization, electrocatalyst, hydrogen

## Abstract

Ni nanowire array electrodes with an extremely large surface area were made through an electrochemical reduction process utilizing an anodized alumina template with a pore length of 320 µm, pore diameter of 100 nm, and pore aspect ratio of 3200. The electrodeposited Ni nanowire arrays were preferentially oriented in the (111) plane regardless of the deposition potential and exhibited uniaxial magnetic anisotropy with easy magnetization in the axial direction. With respect to the magnetic properties, the squareness and coercivity of the electrodeposited Ni nanowire arrays improved up to 0.8 and 550 Oe, respectively. It was also confirmed that the magnetization reversal was suppressed by increasing the aspect ratio and the hard magnetic performance was improved. The electrocatalytic performance for hydrogen evolution on the electrodeposited Ni nanowire arrays was also investigated and the hydrogen overvoltage was reduced down to ~0.1 V, which was almost 0.2 V lower than that on the electrodeposited Ni films. Additionally, the current density for hydrogen evolution at −1.0 V and −1.5 V vs. Ag/AgCl increased up to approximately −580 A/m^2^ and −891 A/m^2^, respectively, due to the extremely large surface area of the electrodeposited Ni nanowire arrays.

## 1. Introduction

Metal nanowires with a high aspect ratio (length/diameter) exhibit shape anisotropy and have a large specific surface area; therefore, they are expected to be used as magnetic and electrocatalytic materials [1,2,3,4,5,6,7]. Ferromagnetic metal nanowires such as Fe, Co, and Ni have large magnetic anisotropy based on their shape; therefore, these metals are considered for new hard magnetic materials that can be used in high-density magnetic recording devices. In comparison to non-ferromagnetic metals such as Cu [8], ferromagnetic metals also exhibit excellent electrocatalytic activity for hydrogen evolution in acidic and alkaline baths [9,10,11,12,13]. For example, Shi et al. reported that the Tafel slope for hydrogen evolution on a copper cathode was 160.6 mV dec^−1^ in an aqueous solution containing 1 M Na_2_SO_4_ and H_2_SO_4_ [8]. Meanwhile, Mohammadi et al. reported that the Tafel slope for hydrogen evolution on a NiCoP cathode was 49 mV dec^−1^ in an aqueous solution containing 1 M KOH, which was an almost identical value to that of the Pt cathode [10]. Among the ferromagnetic metals, Ni nanowires have been extensively investigated because they are relatively inexpensive and exhibit corrosion resistance [1,4,14].

Metallic nanowires can be synthesized by using an electrodeposition technique from an aqueous solution using a nanochannel template [15], a solvothermal method [16], and a molecular beam epitaxy method [17]. Among them, it is well known that the electrodeposition technique using a nanochannel template has an advantage in terms of cost performance because it enables us to produce numerous metallic nanowires at room temperature under atmospheric pressure [18,19,20,21,22]. As a nanochannel template material for the electrodeposition of metals, anodized aluminum oxide (AAO) templates [23,24] and ion track etched polycarbonate templates [25,26] can be utilized. Zhang et al. discovered that Co nanowires were able to be electrodeposited by utilizing an AC electrodeposition technique into AAO nanochannels with pore diameters of 25 nm, 50 nm, and 75 nm. They revealed that the magnetic squareness of the electrodeposited Co nanowires decreased from 0.924 to 0.525 with increasing the pore diameter up to 75 nm [27].

Some researchers have investigated how nickel and nickel alloy nanowires could be synthesized into nanochannels. For example, Thiem et al. reported that CoNiP nanowire arrays could be fabricated into a polycarbonate nanochannel template by utilizing a potentiostatic deposition technique [28]. They discovered that the squareness of the electrodeposited CoNiP nanowires with a diameter of 100 nm and a length of 3 µm was approximately 0.25. Additionally, Vazquez et al. discovered that Ni nanowires could be produced into alumina nanochannel templates by utilizing a pulsed current electrolysis technique [29]. They revealed that the squareness of the electrodeposited Ni nanowires that were 85 nm in diameter and 4 µm in length was around 0.3. Escrig et al. also discovered that Ni nanowires could be synthesized into alumina nanochannel templates by utilizing a potentiostatic electrodeposition technique [30]. They found that the squareness of the electrodeposited Ni nanowires with a diameter of 100 nm and length of 12 µm was around 0.6. They also discovered that the squareness was improved with increasing the aspect ratio.

Most researchers have attempted to refine the pore diameter of AAO templates to obtain ferromagnetic nanowires with a small diameter, which can improve the hard magnetic performance [31,32,33,34,35,36]. If the pore diameter of AAO templates is smaller than several tens of nanometers, it will be quite difficult to realize a uniform pore-filling with electrodeposited metallic crystals due to the limitation of metal ion transportation inside the narrow channels. Hence, we attempted to increase the pore length to realize an extremely large aspect ratio in the present study. However, if the anodic oxidation time is longer than several tens of hours at room temperature, the AAO films will be redissolved in the acidic aqueous solution [37]. Therefore, it is quite difficult to make an AAO template with an extremely large pore length. If we can keep the solution temperature lower than 10 degrees Celsius by utilizing a cool incubator system, the redissolution of the AAO film will be prevented and an AAO template with an extremely large pore length could be synthesized. By utilizing an AAO template with a large aspect ratio, the specific surface area of electrodeposited Ni nanowire arrays will be significantly increased. Hence, the electrocatalytic activity for hydrogen evolution on the electrodeposited Ni nanowire arrays will be also improved.

Therefore, in the present study, we developed AAO templates with a pore length (*L*) of 320 µm, pore diameter (*D*) of 100 nm, and pore aspect ratio (*L*/*D*) of 3200 by keeping the solution temperature lower than ten degrees Celsius by utilizing a cool incubator system during the anodic oxidation process. Subsequently, Ni nanowire arrays with an ultra-high aspect ratio were electrochemically synthesized using the developed AAO templates. The preferential crystal orientation, uniaxial magnetization performance, and electrocatalytic activity for hydrogen evolution were also investigated.

## 2. Materials and Methods

A commercially available aluminum rod (The Nilaco Corporation, Tokyo, Japan, item number: AL-012659, purity: 99%) with a diameter of 10 mm was mechanically polished in the cross-section area using sandpaper (#1500). Subsequently, to achieve a mirror-like surface finish, electrochemical polishing was applied to the cross-section of the aluminum rod in an ethanol solvent with 20 vol.% perchloric acid (Wako Pure Chemical Industries, Ltd., Osaka, Japan, 60–62 mass%). During the mirror-like surface finishing process, the cell voltage was maintained at 50 V for 2 min. Anodic oxidation was then performed in an aqueous solution containing 0.6 mol/L oxalic acid. During the anodic oxidation process, the cell voltage was kept at 90 V for 24 h to generate an anodic oxide layer with a nanochannel structure. After the anodic oxidation process, the thin disc layer of aluminum oxide was exfoliated from the cross-section of the metallic aluminum rod in an ethanol solvent with 50 vol.% perchloric acid to produce an anodized aluminum oxide (AAO) nanochannel template. Subsequently, the AAO nanochannel template was chemically etched in an aqueous solution containing 8 wt.% phosphoric acid at room temperature (25 °C) to remove the barrier layer [33]. After the chemical etching process, a conductive metallic copper layer was sputtered on one side of the AAO nanochannel template to apply as a working electrode for growing Ni nanowire arrays. The nanochannel diameter and length were determined by using a field-emission scanning electron microscope (FE-SEM, JSM-7500FA, JEOL, Tokyo, Japan) and a digital micrometer (MDC-25MX, Mitsutoyo, Yokohama, Japan), respectively.

The nanowires were electrochemically synthesized into the AAO nanochannel template in an aqueous solution containing 0.5 M nickel sulfate and 0.4 M boric acid. The solution pH and temperature were kept at 4.0 and 40 °C, respectively. Before the electrochemical growing process, the AAO nanochannel templates were immersed in an aqueous solution containing 0.5 M nickel sulfate and 0.4 M boric acid under reduced pressure to fill the solution into the nanochannels. A metallic nickel plate was used as a soluble anode while a Ag/AgCl electrode was used as a reference electrode. During the potentiostatic electrochemical reduction, the growing process of Ni nanowires was monitored by measuring the time dependence of the cathodic current. After the electrochemical growing process, the AAO nanochannel templates were removed by dissolving in an aqueous solvent with 5 M NaOH to separate the Ni nanowire arrays from the AAO templates. Subsequently, the morphology of the recovered Ni nanowires was investigated by utilizing a field-emission scanning electron microscope (FE-SEM, JSM-7500FA, JEOL, Tokyo, Japan) and a transmission electron microscope (TEM, JEM-2010-UHR, JEOL Ltd., Tokyo, Japan).

The preferential crystal orientation of the Ni nanowire arrays was analyzed by using X-ray diffraction (XRD, MiniFlex 600-DX, Rigaku Corp., Tokyo, Japan) patterns and electron diffraction (ED) patterns. The uniaxial magnetization performance of the Ni nanowires was evaluated using a vibrating sample magnetometer (VSM, TM-VSM1014-CRO, Tamakawa Co. Ltd., Sendai, Japan) by increasing the magnetic field up to 10 kOe at room temperature. Electrocatalytic activity for hydrogen evolution on the Ni nanowire array electrodes was investigated using a linear sweep voltammogram (LSV, Electrochemical Measurement System, HZ-7000, Hokuto Denko Corp., Tokyo, Japan) in an aqueous solvent with 0.025 M sulfuric acid at 40 °C. During the LSV measurement, a gold wire was used as an insoluble anode while a Ag/AgCl electrode was used as a reference electrode. The cathode potential was linearly swept in the range from −0.5 V to −1.5 V vs. Ag/AgCl.

## 3. Results and Discussion

### 3.1. Fabrication of Anodized Aluminum Oxide (AAO) Template

**Figure 1:**
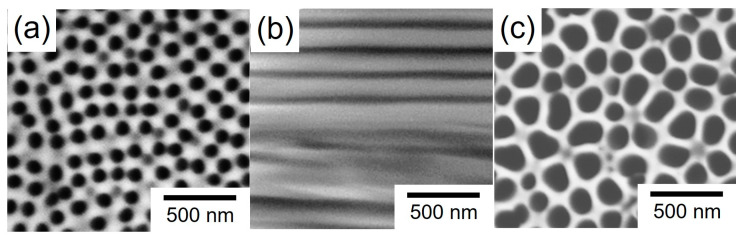
SEM images of an anodic oxidation coating exfoliated from a metallic aluminum surface: (**a**) top view of the film, (**b**) cross-section view of the film, and (**c**) bottom view of the film. The anodization cell voltage was kept at 90 V for 24 h.

Figure 1 shows SEM images of the bottom surface (a), cross-section (b), and top surface (c) of an AAO nanochannel template that was anodically oxidized at 90 V for 24 h. During the anodic oxidation process, the bath temperature was kept at 10 °C to prevent the redissolution of the AAO film in the aqueous solution containing oxalic acid. According to these SEM images, it was confirmed that the AAO nanochannel template had a through-hole structure. Therefore, in the present study, the nanochannel length was assumed to be the same as the AAO film thickness. The film thickness was determined by using a digital micrometer as described in the experimental section. The exfoliated AAO template had a nanochannel diameter (D) of ~100 nm and a nanochannel length (L) of ~320 µm. Hence, the aspect ratio (L/D) of the nanochannels was estimated to be ~3200.

### 3.2. Electrochemical Growth of Ni Nanowires in the AAO Nanochannels

**Figure 2:**
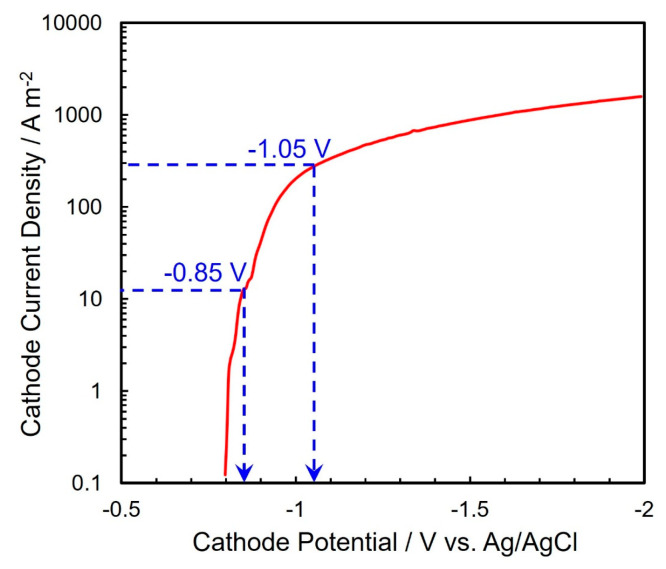
Cathode polarization curve for the electrodeposition of Ni from an acidic aqueous solution.

Figure 2 shows the cathodic polarization behavior for Ni electrochemical growing from an acidic aqueous solvent with nickel sulfate and boric acid. During the measurement, a copper foil was used as a cathode while a nickel plate was used as a soluble anode. In addition, a Ag/AgCl electrode was also used as a reference electrode. The potential sweep rate was fixed at 50 mV s^−1^. The equilibrium potential for Ni/Ni^2+^ can be estimated by the following Nernst Equation (1):

(1) $$E_{Ni}^{eq}=E_{Ni}^{0}+\frac{RT}{2F}ln\frac{\left[{Ni}^{2+}\right]}{\left[{Ni}^{0}\right]}$$

where $E_{Ni}^{eq}$, $E_{Ni}^{0}$, *F*, and *R* represent the equilibrium potential for Ni/Ni^2+^, the standard electrode potential for Ni/Ni^2+^, the Faraday constant (96,485 C mol^−1^), and the gas constant (8.31 J K mol^−1^), respectively, and *T*, [*M^n+^*], and [*M*^0^] denote the absolute temperature, the activity of metal ions, and the activity of electrodeposited metal, respectively.

According to the above Nernst equation, $E_{Ni}^{eq}$ can be determined to be −0.436 V vs. Ag/AgCl at 40 °C bath temperature. As depicted in the cathodic polarization behavior (Figure 2), however, the cathodic current rises up at a potential of around −0.80 V vs. Ag/AgCl. Usually, the electrochemical reduction of iron group metal ions such as Fe^2+^, Co^2+^, and Ni^2+^ proceeds accompanying a kinetic overpotential based on the multi-step electrochemical reduction mechanism via hydroxide ions as per the following Equations (2)–(4) [38]:

Ni^2+^ + OH^−^ = NiOH^+^ (2)

NiOH^+^ + e^−^ = [NiOH]_ad_ (3)

[NiOH]_ad_ + e^−^ = Ni + OH^−^ (4)

Therefore, this increase in the cathodic current (Figure 2) seems to correspond to the electrodeposition current of Ni^2+^ ions. According to Figure 2, the following Tafel Equation (5) can be applied in the cathode current density range from 1 A/m^2^ to 100 A/m^2^.

*η*_c_ = *a* + *b* log *i*_c_ (5)

where *η*_c_, *i*_c_, *a*, and *b* represent the cathodic overpotential, cathode current density, Tafel constant, and Tafel slope, respectively. The Tafel slope, *b*, can be also described by the following Equation (6):

(6) $$b=\frac{2.303RT}{\alpha zF}$$

Here, *α* and *z* correspond to the charge transfer coefficient and number of electrons exchanged, respectively. If the electrodeposition process of Ni is dominated by the above Equation (4), *α* and *z* can be assumed as 0.5 and 1, respectively. Hence, the Tafel slope, *b*, can be estimated as 0.124. This theoretical value corresponds well with the experimental value (*b* = 0.13) which was determined from the Tafel plot in Figure 2 in the cathode current density range from 1 A/m^2^ to 100 A/m^2^.

Moreover, with increasing the cathode current density to more than 200 A/m^2^, the cathode potential polarized significantly because the rate-determining stage shifted from the charge-transfer stage (Equation (4)) to the mass-transfer stage of Ni^2+^ ion migration and diffusion. In this cathode current density range, the cathode current efficiency will be decreased due to hydrogen evolution which is caused by the decomposition of water solvent. This side reaction will result in the formation of powder-like or dendrite-like Ni deposits. Furthermore, the Ni^2+^ ion diffusion coefficient in AAO nanochannels will be smaller than that on a flat cathode. Therefore, in the present study, the cathode potential for the electrochemical growth of Ni nanowire arrays was fixed to the range from −0.85 V to −1.05 V vs. Ag/AgCl.

**Figure 3:**
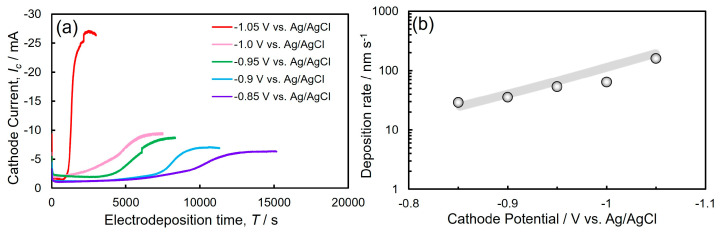
(**a**) Time dependence of cathode current during the electrodeposition of Ni nanowires from the sulfuric acid bath. (**b**) Effect of cathode potential on the deposition rate of Ni nanowires.

Figure 3a depicts the time dependence of the cathode current during the Ni nanowires growth utilizing a potentiostatic electrodeposition technique. At the first stage, the observed current value was almost constant due to the Ni nanowires’ homogeneous growth in the AAO nanochannels. After the first stage, the cathode current value increased rapidly at the electrodeposition time of several thousand seconds. Usually, the cathodic current will be in proportion to the cathode surface area. Hence, this drastic enhancement in the cathode current seemed to be induced by the formation of film-like Ni deposits on the outside of the nanochannels. Furthermore, with an increase in the electrochemical reduction time, the cathode current asymptotically reached a stable value. At this final stage, the AAO template surface seemed to be completely covered with the electrodeposited Ni film and the cathode surface area seemed to reach a maximum value. However, in the final stage at −1.05 V (red line in Figure 3a), the cathode current slightly decreased. This seems to be owing to the formation of cracks, which can be induced from the internal stress of the electrodeposited Ni film. According to the duration of the first stage in Figure 3a, the crystal growth rate of electrodeposited Ni nanowires was determined by dividing the length of the AAO nanochannels by the growth time of the Ni nanowires in the AAO nanochannels. Figure 3b depicts the cathode potential dependence on the growth rate of Ni nanowires. With increasing the cathodic overvoltage, the growth rate of Ni nanowires decreased exponentially. Ni nanowires were electrodeposited at a growth rate ranging from 20 to 70 nm/s, which was significantly faster than that achieved by a conventional sputtering process (approximately 1 nm/s) [39].

### 3.3. Crystal Texture of Electrodeposited Ni Nanowires

**Figure 4:**
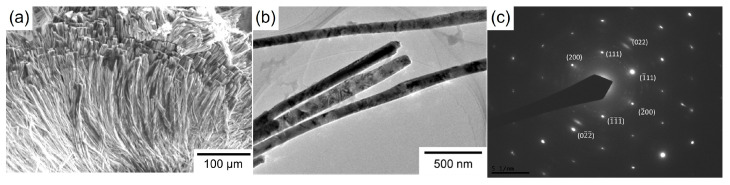
(**a**) SEM image of electrodeposited Ni nanowire array. (**b**) TEM image (brightfield image) of electrodeposited Ni nanowires. (**c**) Electron diffraction patterns of electrodeposited Ni nanowires.

Figure 4a depicts an SEM image of an electrochemically grown Ni nanowire array that was recovered from a dissolved AAO template. According to the SEM image, the numerous Ni nanowires aggregated each other due to their ultra-high aspect ratio of more than 3000.

Figure 4b,c show a TEM brightfield image and an ED pattern of the electrochemically grown Ni nanowires. The TEM image reveals that the diameter of the electrodeposited Ni nanowires is around 100 nm, which is almost identical to the pore diameter of the AAO template. It was also confirmed that the Ni nanowires that were grown at the potential of −0.90 V vs. Ag/AgCl consisted of an fcc-Ni phase due to the ED spot pattern arrangement. In particular, spots deriving from fcc-Ni (111) were clearly observed.

**Figure 5:**
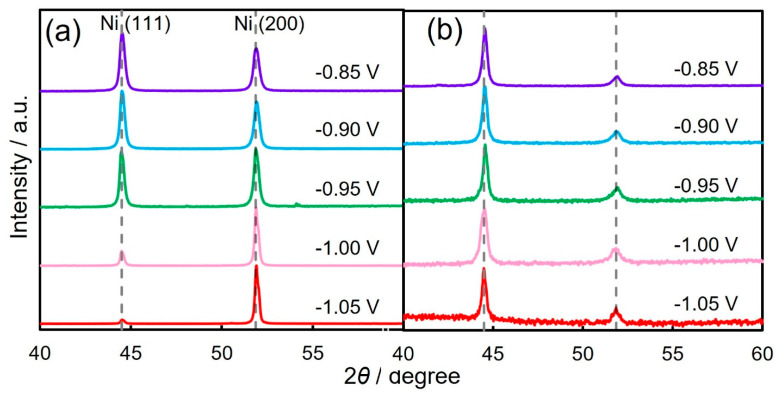
X-ray diffraction patterns of Ni thin films (**a**) and Ni nanowire arrays (**b**) that were electrodeposited at −0.85 V, −0.90 V, −0.95 V, −1.00 V, and −1.05 V.

Figure 5a depicts the XRD profiles of Ni thin films that were electrochemically grown at cathode potentials of −0.85 V, −0.90 V, −0.95 V, −1.00 V, and −1.05 V. The electrodeposition conditions such as bath composition and film thickness for the Ni thin films were the same as those for the Ni nanowires. In these XRD patterns, the peak (2*θ* = 44.5°) that was derived from fcc-Ni (111) was observed clearly when the sample was electrodeposited at a noble potential range, while the peak (2*θ* = 51.8°) that was derived from fcc-Ni (200) was also observed apparently as the sample was grown at a less noble potential range [40]. In contrast, according to the XRD pattern that was obtained from the electrodeposited Ni nanowire arrays, the fcc-Ni (111) was strongly oriented as shown in Figure 5b. This tendency corresponds well to the result which was obtained from the electron diffraction pattern during the TEM observation (Figure 4c).

On the basis of the above XRD patterns, the crystal texture coefficients ${TC}_{\left(hkl\right)}$ of the electrodeposited Ni nanowire arrays were calculated using the following Harris equation [41,42]:

(7) $${TC}_{\left(hkl\right)}=\frac{\frac{I_{hkl}^{i}}{I_{hkl}^{0}}}{\frac{1}{N}\times \sum_{j=1}^{N}\left(\frac{I_{hkl}^{j}}{I_{hkl}^{0}}\right)}$$

where $I^{0}$ denotes the intensity of each peak in the XRD profile that was obtained from a standard Ni powder, $I^{i}$ denotes the intensity of each peak in the XRD profile that was obtained from the electrodeposited Ni sample, (*h k l*) corresponds to each lattice plane of the Ni crystal, and *N* is the number of observed diffraction profiles.

**Figure 6:**
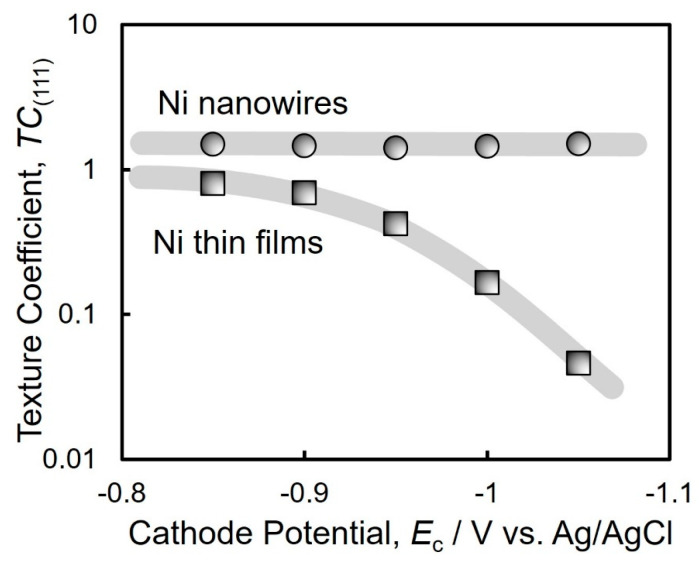
Effect of cathode potential on the texture coefficients ${TC}_{\left(111\right)}$ of Ni thin films and Ni nanowire arrays.

Figure 6 depicts the cathode potential dependence on the texture coefficients ${TC}_{\left(111\right)}$ of electrodeposited Ni thin films (a) and electrodeposited Ni nanowire arrays (b). The ${TC}_{\left(111\right)}$ of the Ni thin films decreased down to 0.046 with shifting the potential to −1.05 V vs. Ag/AgCl. This is because the overvoltage was increased by shifting the potential to a less noble direction during the electrodeposition. This result is consistent with Pangarov’s theory [43,44,45]. In contrast, the ${TC}_{\left(111\right)}$ of the Ni nanowires kept a stable value higher than 1.4 over the wide cathode potential range.

### 3.4. Magnetic Properties of Electrochemically Grown Ni Nanowires

**Figure 7:**
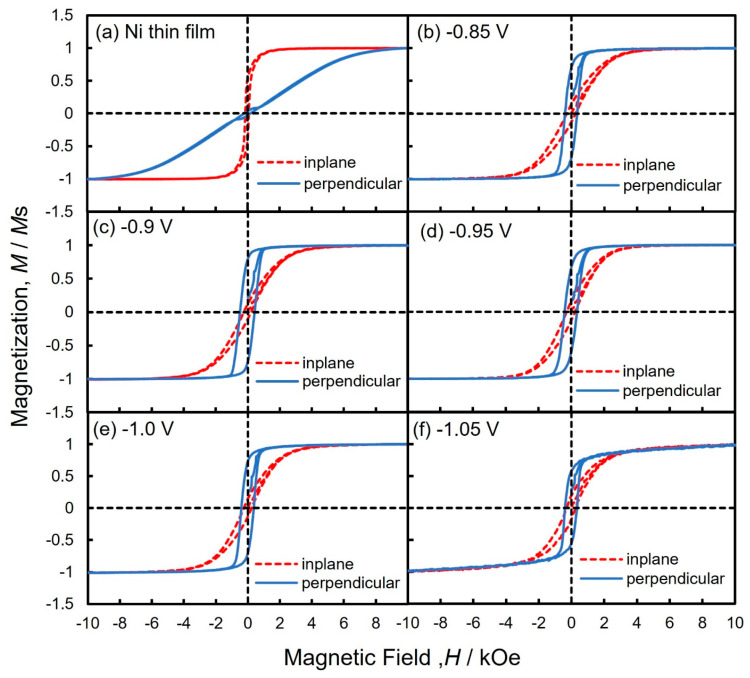
Magnetic hysteresis loops of Ni thin films and Ni nanowire arrays that were electrodeposited at each cathode potential. Magnetic field was applied in the in-plane direction and perpendicular to the film plane.

Figure 7 depicts the magnetic hysteresis loops of Ni thin films and Ni nanowire arrays that were electrodeposited in the nanochannels of AAO films. The magnetic hysteresis loops were obtained when an external magnetic field was applied in an in-plane direction (dashed line) and perpendicular direction (solid line) to the thin film and AAO film plane. The perpendicular direction corresponds to the axial direction of Ni nanowires. As shown in the blue solid lines in Figure 7b–f, Ni nanowires were magnetized spontaneously in the axial direction and exhibited uniaxial magnetic anisotropy. Thus, the shape magnetic anisotropy which is based on the high aspect ratio of the Ni nanowires was confirmed.

**Figure 8:**
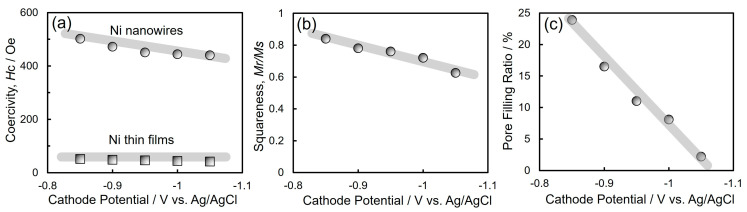
Effect of cathode potential on coercivity (**a**), squareness (**b**), and pore filling ratio (**c**) of electrodeposited Ni nanowire arrays.

Figure 8 shows the cathode potential dependence on the coercivity (a), squareness (b), and pore filling ratio (c) of the electrodeposited Ni nanowire arrays. As shown in Figure 8a, the coercivity of the electrodeposited Ni thin films was only 45 Oe [46]. On the contrary, the coercivity of the electrodeposited Ni nanowire arrays increased up to 502 Oe. On the basis of the XRD patterns in Figure 5b, the electrodeposited Ni nanowire arrays exhibited a preferential crystal growth direction in fcc-Ni [111] which corresponds to the easy magnetization direction of fcc-Ni crystal. Therefore, the enhancement in the coercivity of electrochemically grown Ni nanowires with the preferential crystal growth direction in [111] seems to be caused by the consistency between the magneto-crystalline anisotropy and shape magnetic anisotropy of Ni nanowires. As shown in Figure 8b, the squareness of electrochemically grown Ni nanowires increased with decreasing the cathodic overpotential and reached up to 0.84 at the potential of −0.85 V vs. Ag/AgCl. Escrig et al. revealed that the squareness of electrodeposited Ni nanowires increased with increasing the aspect ratio and reached around 0.7 in a sample with an aspect ratio of 240 (50 nm in diameter and 12 µm in length) [30]. Neetzel et al. synthesized electrochemically grown Fe nanowire arrays with a diameter (*D*) of 85 nm, average length (*L*) of 60 µm, and average aspect ratio (*L/D*) of approximately 706 [36]. They revealed that the electrodeposited Fe nanowire arrays exhibited squareness (*Mr/Ms*) and coercivity (*Hc*) values of approximately 0.59 and 550 Oe, respectively. Fan et al. reported that they electrodeposited Co/Cu multilayered nanowires with an average diameter (*D*) of 60 nm, a length (*L*) of 7 µm, and an aspect ratio (*L/D*) of approximately 117 [47]. They discovered that the coercivity (*Hc*) and squareness (*Mr/Ms*) of the multilayered nanowires were approximately 570 Oe and 0.15, respectively. Usually, electrodeposited iron group metal films are strongly affected by a demagnetizing field when an external magnetic field is applied in the perpendicular direction to the film surface. On the contrary, for cylindrical iron group metal nanowires with a large aspect ratio, the demagnetization factor becomes small when an external magnetic field is applied in the axial direction. The demagnetizing field, $H_{d}$, can be given by the following Equation (8):

(8) $$H_{d}=\left(\frac{N_{d}}{\mu_{0}}\right)\times M$$

where $N_{d}$ represents the demagnetization factor, $\mu_{0}$ corresponds to the magnetic permeability $\left(4\pi \times 10^{-7}\left[H/m\right]\right)$, and *M* means the magnetization. Additionally, $N_{d}$ can be expressed by Equation (9) as a function of the aspect ratio, *k* =*L/D* (*L*: length of nanowire; *D*: diameter of nanowire):

(9) $$N_{d}=\frac{1}{k^{2}-1}\left\{\frac{k}{\sqrt{k^{2}}-1}\ln\left(k+\sqrt{k^{2}-1}\right)-1\right\}$$

According to the above Equation (9), the demagnetization factor *N*_d_ will decrease down to 1.7 × 10^−5^ from 4.3 × 10^−4^ when the aspect ratio *k* increases up to 600 from 100. Furthermore, *N*_d_ will decrease down to 8.6 × 10^−7^ when *k* increases up to 3000. Thus, the demagnetization factor decreases as the aspect ratio increases. On the basis of Equation (8), the demagnetizing field will decrease with a decrease in the demagnetization factor. Therefore, the nanowires will be magnetized easily in the axial direction when the aspect ratio increases. This uniaxial magnetization performance will enhance the hard magnetic properties such as squareness and coercivity. As depicted in Figure 8b, the squareness improved as the potential shifted to the electrochemical noble region. This improvement in the squareness seems to be attributed to the increase in the pore filling ratio as shown in Figure 8c. If the cathodic overvoltage is decreased, the throwing power will be improved because the rate-limiting process during the electrodeposition will become the charge transfer process from the mass transfer process, such as the migration of metal ions. Therefore, the average aspect ratio of electrodeposited Ni nanowires seems to be enhanced by decreasing the cathodic overvoltage.

### 3.5. Electrocatalytic Performance for Hydrogen Evolution on the Electrodeposited Ni Nanowire Arrays

**Figure 9:**
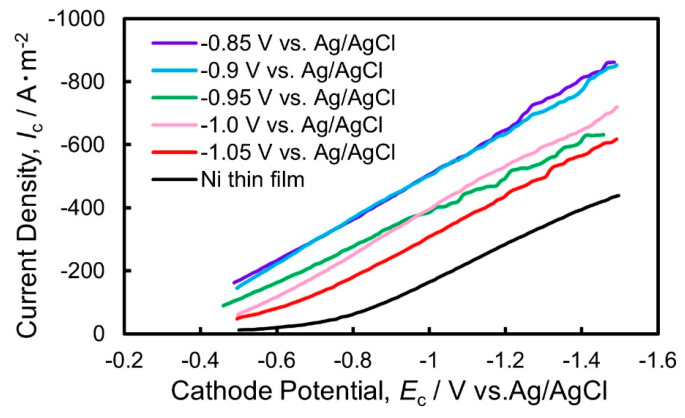
Cathodic polarization curves for hydrogen evolution on the electrodeposited Ni nanowire arrays to investigate the electrocatalytic performance.

Figure 9 depicts the polarization curves for hydrogen evolution reaction on the electrodeposited Ni thin films and Ni nanowire arrays in an aqueous solvent with sulfuric acid. According to Figure 9, the hydrogen evolution reaction occurred at the potential of approximately −0.5 V vs. Ag/AgCl on the Ni thin film electrode whereas it occurred at the potential of approximately −0.3 V vs. Ag/AgCl on the Ni nanowire array electrodes. Hence, the minimum hydrogen overvoltage is estimated to be around 0.3 V on the electrodeposited Ni thin films whereas it is estimated to be around 0.1 V on the Ni nanowire array electrodes. This reduction in the minimum hydrogen overvoltage seems to be caused by the enhancement of the specific surface area on the electrodeposited Ni nanowire array electrodes.

The cathode current density for hydrogen evolution reaction on the Ni nanowire array electrode with a pore filling ratio of 23.9% was approximately −580 A/m^2^ at the potential of −1.0 V vs. Ag/AgCl, whereas that on the Ni thin film electrode was around −170 A/m^2^. Lee et al. discovered that they could fabricate Ni nanowire arrays of 20 µm in average diameter (*D*), 200 µm in length (*L*), and approximately 100 in aspect ratio (*L/D*) [4]. They revealed that the cathode current density at the potential of −1.0 V vs. Ag/AgCl was approximately −50 A/m^2^. Furthermore, Nie et al. reported that they could synthesize Co-Ni nanowire arrays of 60 nm in average diameter (*D*), 60 µm in length (*L*), and approximately 1000 in aspect ratio (*L/D*) [7]. They found that the cathode current density at the potential of −1.0 V vs. Ag/AgCl was around −145 A/m^2^.

In the present study, the cathode current density at −1.5 V was approximately −891 A/m^2^, which was superior to the above-mentioned previous studies due to the increased aspect ratio of the Ni nanowire arrays. If the Ni nanowires which were obtained in the present study had an ideal array structure, the specific surface area would be around 1965 times larger than that of a Ni thin film. However, as shown in Figure 4a, the bundle structure which was caused by the aggregation between the nanowires seemed to inhibit the enhancement in the specific surface area. Further improvements in the electrocatalytic performance will be realized if we can precisely optimize the nanowire array structure, such as the diameter, length, and inter-wire distance.

## 4. Conclusions

Ni nanowire arrays with an ultra-high density were synthesized by utilizing a potentiostatic electrodeposition technique into AAO nanochannels with a length of 320 µm, diameter of 100 nm, and aspect ratio of 3200. The Ni nanowire arrays had a textured structure with a strong orientation of fcc-Ni [111] in the axial direction regardless of the electrodeposition potential. The uniaxial magnetization behavior was confirmed in the axial direction of the electrodeposited Ni nanowire arrays. Additionally, the magnetic performance of the Ni nanowires improved by increasing the aspect ratio. The squareness and coercivity increased up to 0.8 and 550 Oe, respectively. Furthermore, regarding the electrocatalytic reaction for hydrogen evolution on the Ni nanowire array cathode, the minimum hydrogen overvoltage decreased down to 0.1 V by applying the nanowire array electrode. The cathode current density for the catalytic reaction during hydrogen evolution increased up to around −580 A/m^2^ at −1.0 V and −891 A/m^2^ at −1.5 V vs. Ag/AgCl. The improvement could be derived from the enhancement in the specific surface area of the electrodeposited Ni nanowire arrays that had an ultra-large aspect ratio.

## Data Availability

Datasets generated during the current study are available from the corresponding author on reasonable request.
